# Sustained ppGpp production underpins months-long survival of a bacterium in growth arrest

**DOI:** 10.1128/mbio.01079-26

**Published:** 2026-06-18

**Authors:** Elizabeth M. Fones, Liang Yin, Dale Whittington, Caroline S. Harwood

**Affiliations:** 1 Department of Microbiology, University of Washington 312771, Seattle, Washington, USA; 2 Department of Medicinal Chemistry, University of Washington 312751, Seattle, Washington, USA; University of California, Berkeley, Berkeley, California, USA

**Keywords:** stationary phase, starvation, stringent response, ppGpp, cellular survival, *Rhodopseudomonas*, phototrophy

## Abstract

When growing bacteria start to reach stationary phase, the nucleotide guanosine tetra-phosphate (ppGpp) accumulates intracellularly and regulates the transition of cells from growth to growth arrest. Because commonly studied bacteria remain viable in stationary phase only briefly under laboratory conditions, the role of ppGpp in sustaining long-term bacterial survival after growth arrest has not been widely studied. *Rhodopseudomonas palustris* strain CGA009 is a phototrophic alpha-proteobacterium that survives under anaerobic conditions for months when not growing due to carbon starvation if provided light. When we quantified intracellular ppGpp in growing and growth-arrested *R. palustris*, we found that it was undetectable in growing cells but accumulated to about 100 µM when cells ran out of carbon and entered the stationary phase. These elevated levels of ppGpp were maintained over a 60-day period of growth arrest. Intracellular GTP was 100–200 µM in growth-arrested cells, and ATP was at 2–4 mM. ppGpp had global effects on gene expression, with over half of the genes in the *R. palustris* genome being activated or repressed by ppGpp in stationary phase cells. These results suggest that, in addition to its known role in facilitating the transition of bacteria from growth to stationary phase and accompanying stress responses, ppGpp is important for prolonging bacterial survival in stationary phase.

**IMPORTANCE:**

The molecular basis for long-term survival of starved bacteria is not well understood. The bacterium *Rhodopseudomonas palustris* has a metabolism in which it survives in growth arrest for months, as long as it can generate ATP by photophosphorylation. This allowed us to determine if the nucleotide ppGpp, produced by bacteria in response to nutrient starvation, plays a role in supporting the viability of non-growing bacteria for long periods. We found that ppGpp accumulated as *R. palustris* entered growth arrest, and its intracellular levels were maintained for 60 days. ppGpp regulated the expression of over half the genes in the *R. palustris* genome in stationary phase cells. This work expands our concept of the effects of ppGpp on bacterial physiology to encompass an important role in long-term bacterial starvation-survival and longevity.

## INTRODUCTION

The canonical bacterial growth curve includes a lag phase where cells start to grow, a logarithmic (log) phase where cells grow at their maximal rate, and a stationary phase in which cells transition to a non-growing state as they run out of nutrients. We know that growth-arrested bacteria can stay alive for variable amounts of time in natural environments depending on the species and their environmental circumstances (1, 2), but we have a relatively poor understanding of the molecular processes that are responsible for bacterial longevity in growth arrest. It is important to better understand this part of the bacterial life cycle for several reasons. As one example in the arena of human health, so-called “persister” cells that are not growing or growing very slowly can make up a small but significant percentage of cells in infections. This subpopulation can be resistant to antibiotics that target processes including cell division and DNA replication that are active only in growing cells. Uncovering molecular mechanisms of bacterial persistence is an active area of research aimed at developing more efficacious treatments for infections (3). In fact, (p)ppGpp, the topic of this paper, has been implicated in driving persistence in some bacteria (4, 5). In terms of biotechnological applications, bacteria in growth arrest are ideal for use as biocatalysts because, since they are not required to devote resources to growth, they can be used to make larger quantities of desired products (6). As a final example, bacteria in many environments, ranging from deserts to oceans, are known to be growing slowly or not at all, and this can hinder their identification and use for basic and bioprospecting purposes. Methods to identify members of this microbial dark matter have focused on bacteria and archaea that are metabolically active or stimulated to be active (7). Knowledge of proteins or biomarkers indicative of growth-arrested bacteria could be helpful for broadening current approaches of metagenomic identification to include quiescent as well as active microbes.

**Fig 1:**
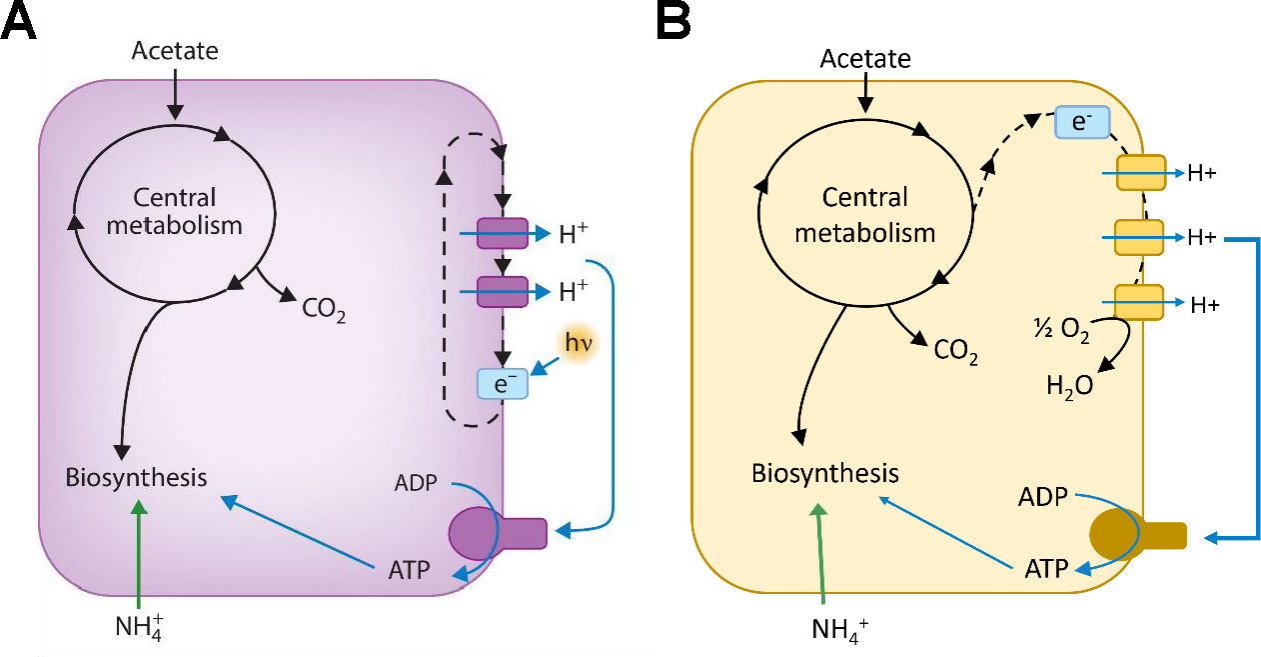
*Rhodopseudomonas palustris* metabolism. During anaerobic growth in light, *R. palustris* generates ATP from light by cyclic photophosphorylation. It uses an organic carbon source like acetate to build biomass. In this growth mode, carbon utilization and energy generation are independent from one another (**A**). During aerobic growth, *R. palustris* uses organic carbon sources like acetate to build biomass and obtain energy from the oxidation of organic carbon coupled to the reduction of oxygen by oxidative phosphorylation (**B**). In this circumstance, carbon starvation also leads to ATP depletion. Panel **A** is adapted from Pechter et al. (16).

Bacteria that have served as excellent laboratory models for studies of the molecular basis of growth tend to survive with full viability for only hours or days in the stationary phase of the growth curve, and this has hampered molecular studies of non-growing cells. We have been developing the metabolically flexible alpha-proteobacterium, *Rhodopseudomonas palustris* strain CGA009 (8, 9), as a model organism for studies of bacterial longevity in growth arrest because this microbe can maintain nearly 100% viability for weeks to months in stationary phase when incubated anaerobically in light (10) (Fig. 1). Factors underpinning the extraordinary longevity of *R. palustris* include sustained protein synthesis at reduced levels and maintenance of high levels of ATP throughout growth arrest via cyclic photophosphorylation (10, 11). Production of guanosine tetraphosphate (ppGpp) is also critical for *R. palustris* longevity (11). ppGpp promotes the expression of genes for stress responses in bacteria and plays a universal role in downregulating ribosome maturation and translation, both of which help cells conserve resources as they run out of nutrients (1, 12–15).

**Fig 2:**
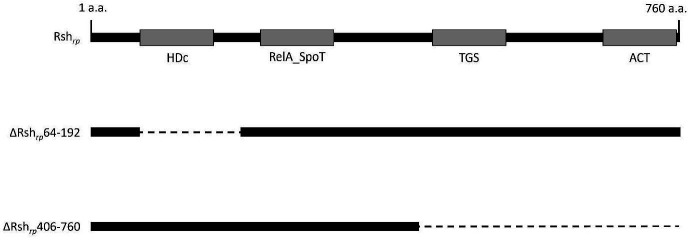
Strains with deletions in the Rsh*_rp_* gene. The ΔRsh*rp*64-192 (ppGpp°) strain was made by deleting the sequence encoding amino acids (a.a.) 64–192 from the *R. palustris* gene RPA2963 (11). The ΔRsh*rp*406-760 strain was constructed by deleting the sequence encoding amino acids 406–760 from the C-terminal regulatory domain (regions TGS and ACT) from Rsh*rp*. This figure is reproduced from reference 11.

Like other alpha-proteobacteria, *R. palustris* has a single bifunctional RelA/SpoT enzyme (RPA2963), that is responsible for both the synthesis of (p)ppGpp from GTP/GDP and ATP and its hydrolysis. We designated the gene encoding this enzyme *rsh_rp_*. In earlier work, we carried out a Tn-seq screen that identified *rsh_rp_* as a longevity gene, which we define as a gene that is not required for growth but is required for survival in growth arrest (16). In follow-up experiments, we validated *rsh_r_*_p_ as a longevity gene by showing that a partial deletion mutant survived poorly in growth arrest. The partial deletion mutant, missing a region corresponding to amino acids 64–192 (Δ*rsh*_rp64-192_), is missing most of the hydrolysis domain of Rsh_rp_ (Fig. 2). We were surprised to find that the Δ*rsh*_rp64-192_ strain did not produce detectable (p)ppGpp during growth or after growth arrest, and one explanation for this is that deletion mutation destabilizes the structure of the Rsh enzyme such that its ability to synthesize (p)ppGpp is also compromised (11). Here, we designate Δ*rsh*_rp64-192_ as the ppGpp° strain. We found that the ppGpp° strain grew about 70% as fast as its wild-type parent and survived poorly in growth arrest (11).

We hypothesized that because of its known role in downregulating protein synthesis in bacteria, ppGpp would need to be constantly present at a relatively elevated level in growth-arrested *R. palustris* to help it preserve cellular resources to support longevity. To test this, we measured levels of ppGpp, ATP, and GTP in growth-arrested *R. palustris* over weeks and months. We focused on ppGpp because *R. palustris* accumulates it in greater amounts than pppGpp. Like other bacteria, *R. palustris* encodes a guanosine-5′-triphosphate, 3′-diphosphate pyrophosphatase (RPA2195) predicted to rapidly convert pppGpp to ppGpp (17). We also measured effects of ppGpp on gene expression after the onset of growth arrest.

## RESULTS

### Conditions of growth arrest

Our routine conditions for establishing growth arrest were to grow *R. palustris* in minimal salts medium with 20 mM acetate anaerobically in light (Fig. 1A). We assigned as day 0 (referred to here as D0) of growth arrest the day at which the OD_660_ of cultures stopped increasing. We measured nucleotides in cultures at day 0 (D0), day 1 (D1), day 6 (D6), day 20 (D20), and day 60 (D60) post-growth arrest, depending on the experiment.

### Nucleotide levels and viability of the *R. palustris* ppGpp° strain in growth and growth arrest

**Fig 3:**
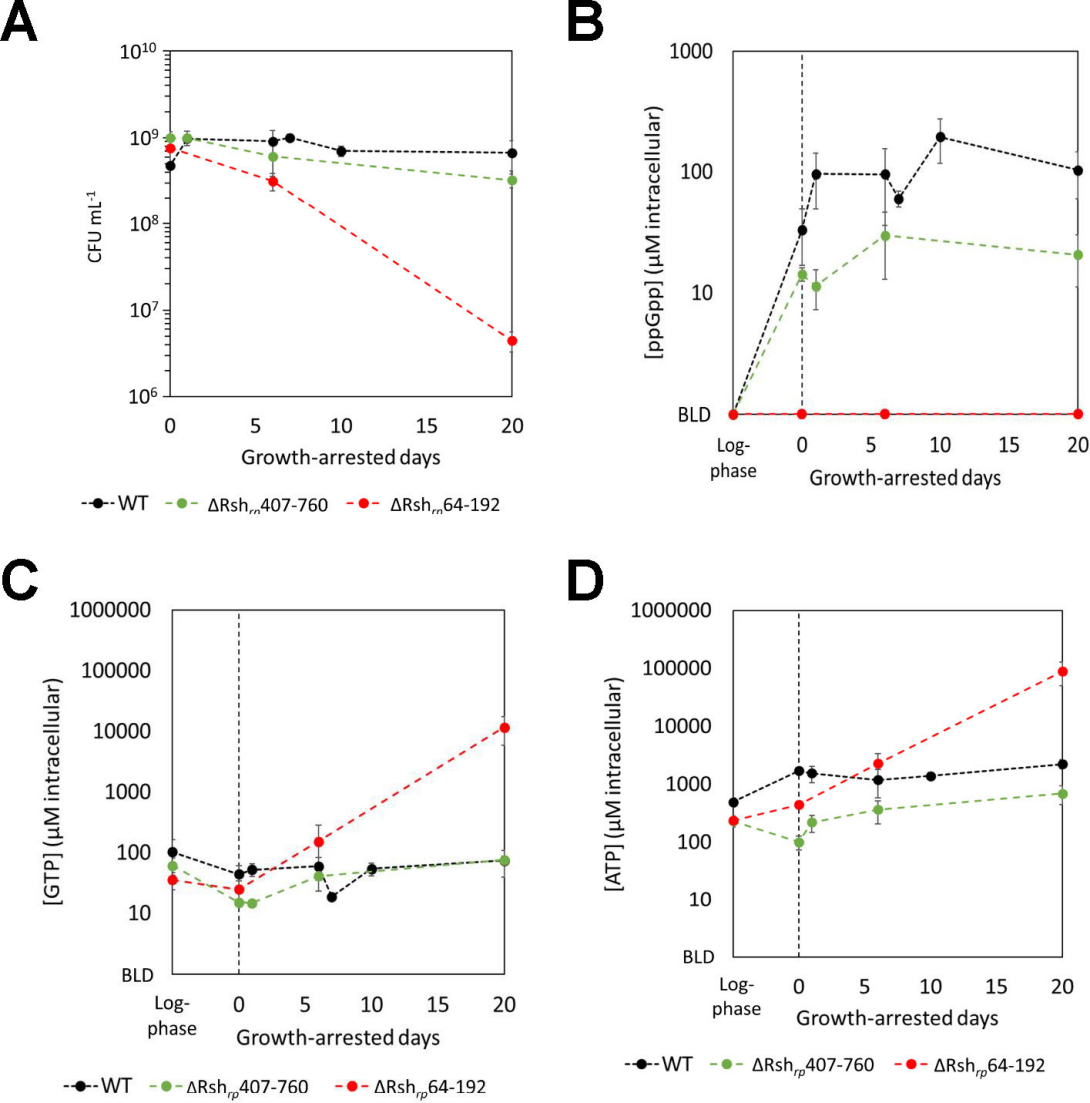
Cell viabilities (**A**) and intracellular concentrations of ppGpp (**B**), GTP (**C**), and ATP (**D**) of wild type (WT), a Δ*rsh_rp_*64–192 strain lacking a part of the predicted N-terminal hydrolase domain of *rsh_rp_,* and a Δ*rsh_rp_*407–760 strain lacking the C-terminal regulatory domain of *rsh_rp_* were compared in growth and growth arrest. Cells were grown anaerobically in light with 20 mM acetate and entered growth arrest at day 0, due to acetate depletion. Cultures were harvested and nucleotides measured by UPLC MS/MS and then normalized to cell number and size as described in Materials and Methods. BLD, below the limit of reproducible detection.

In initial experiments, we compared the viabilities in growth arrest of the ppGpp° strain, the wild type (WT), and a second mutant strain that we constructed (Δrsh_rp407-760_) that lacks the predicted C-terminal regulatory region of Rsh_rp_ (Fig. 2) (15, 18). As reported previously (11), the ppGpp° strain lost over 100-fold viability over 20 days of growth arrest. However, WT *R. palustris* and the *rsh_rp_* C-terminal deletion mutant retained almost 100% viability over the same period (Fig. 3A). The amount of ppGpp in all three of the strains in the log phase of growth was below levels that we could accurately quantify. At D0, intracellular ppGpp levels had risen to 100 and 80 µM in WT and the *rsh_rp_* C-terminal deletion mutant, respectively, but remained undetectable in the ppGpp° strain (Fig. 3B). All three strains had similar intracellular GTP levels of 50–100 µM during log phase and at D0. Levels of ATP in the C-terminal deletion strain were about 0.5 mM during growth and 0.8 mM during growth arrest compared to 0.8 mM during growth and 1 mM during growth arrest for the WT (Fig. 3C and D). At D20 of growth arrest, intracellular ppGpp was about 100 and 50 µM in WT and the C-terminal deletion strain but undetectable in the ppGpp° strain (Fig. 3B). Surviving cells of the ppGpp° strain had highly elevated levels of intracellular ATP (100 mM) and GTP (10 mM) compared to the WT and C-terminal deletion strain (Fig. 3C and D). ppGpp is a potent inhibitor of enzymes for purine biosynthesis and salvage in *Escherichia coli* and *Bacillus subtilis* (19–22). Many of these enzymes have homologs in *R. palustris*, and their lack of inhibition by ppGpp likely explains why the ppGpp° strain synthesized such high levels of ATP and GTP.

### Recovery from growth arrest

**Fig 4:**
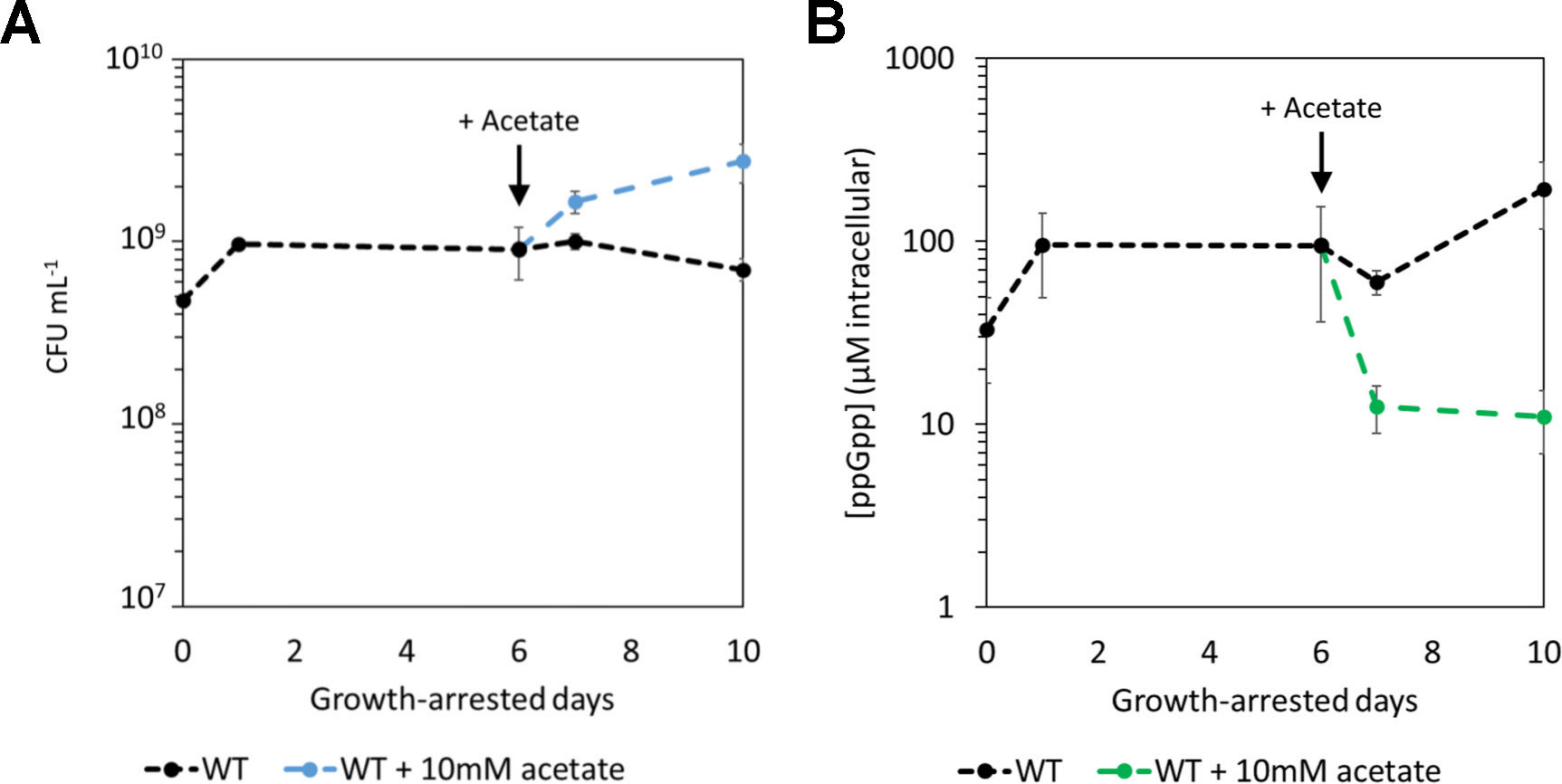
Response of growth-arrested *R. palustris* to addition of 10 mM acetate. *R. palustris* cells were grown anaerobically in light until they entered growth arrest due to depletion of the carbon source, 20 mM acetate. Three replicate vials per timepoint were then amended with an additional 10 mM sodium acetate (blue circles) at day 6 of growth arrest and three replicate vials per time point continued in growth arrest without added carbon (black circles). ppGpp concentrations and viable cell densities were quantified (panels **A and B**, respectively) 7 and 10 days after cells had originally entered growth arrest due to carbon limitation.

We do not know how the *R. palustris* Rsh enzyme is regulated, but since growing cells have very low levels of ppGpp, we predicted that the elevated levels of ppGpp seen in growth-arrested cells would drop when cells started to grow again. Consistent with this, when cells that had been in growth arrest for 6 days started to grow after the addition of 10 mM acetate to the culture (Fig. 4A), there was a corresponding 10-fold drop in intracellular ppGpp (Fig. 4B).

### *R. palustris* wild type grown and incubated anaerobically in light maintained constant ppGpp levels for 60 days post-growth arrest

**Fig 5:**
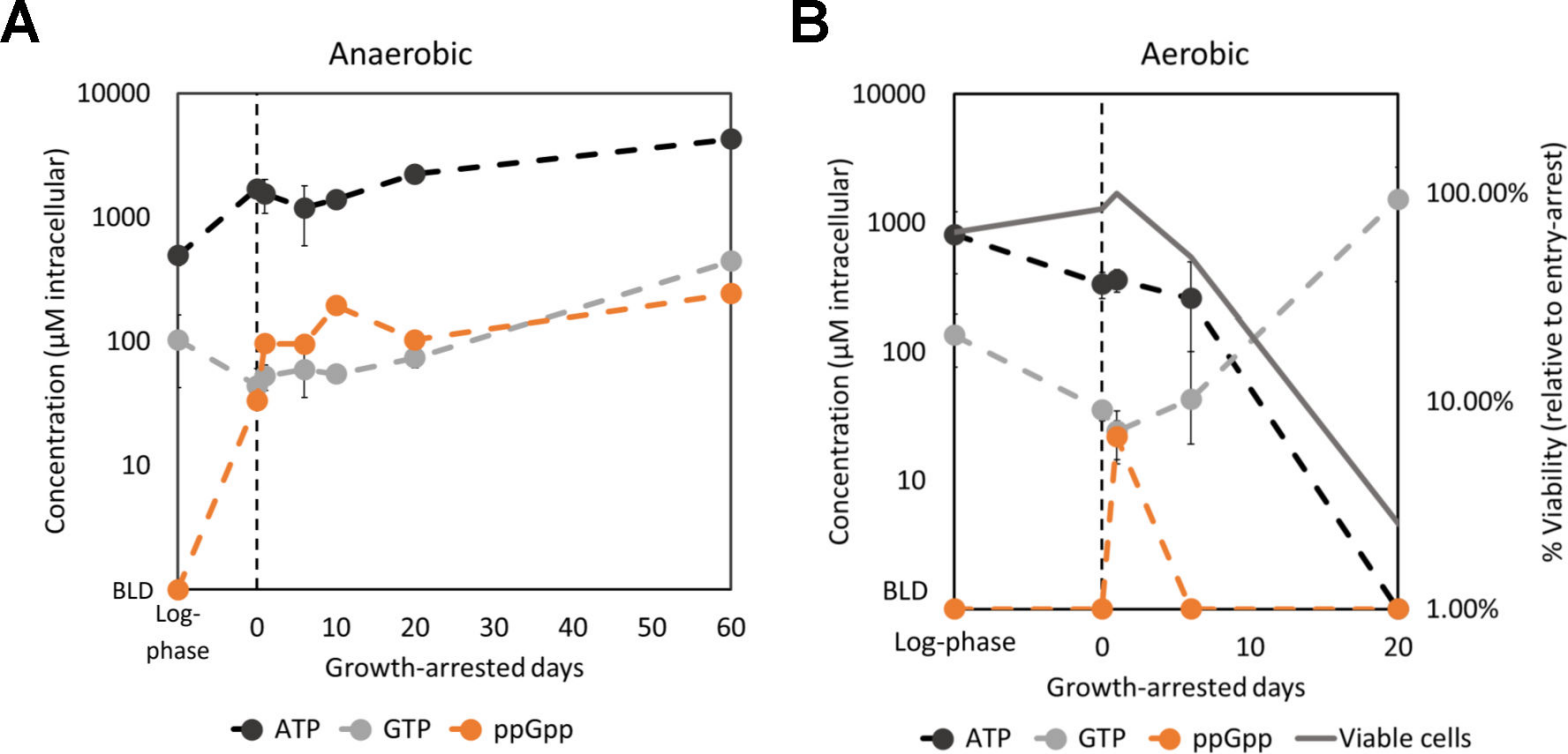
Cell viabilities and intracellular nucleotide concentrations of wild-type *R. palustris* grown anaerobically in light to stationary phase and then incubated in the same conditions in growth arrest for 60 days (**A**) and grown aerobically in dark to stationary phase and incubated in the same conditions in growth arrest for 20 days (**B**). Log-phase samples were collected in early-mid exponential phase (OD_660_ = 0.3–0.4 for anaerobic cultures before plateauing at OD_660_ ~0.8. OD_660_ = 0.1–0.2 for aerobic cultures before plateauing at OD_660_ ~0.4). Cell viability was determined by plating CFU. BLD = below the limit of reproducible detection.

Our longest starvation survival experiments were for a period of 60 days after growth arrest. When we followed nucleotide levels in WT over this time interval, we found that intracellular ppGpp and GTP were maintained at between 100 and 200 µM throughout, and ATP levels were maintained at between 2 and 4 mM (Fig. 5A).

### *R. palustris* wild type grown and incubated aerobically had transiently elevated ppGpp following growth arrest

*R. palustris* grows aerobically as a heterotroph as well as anaerobically as a phototroph. In aerobic growth, cells generate ATP from acetate and other carbon sources by oxidative phosphorylation (Fig. 1B). When aerobically grown cells entered stationary phase due to acetate depletion, intracellular ppGpp increased to 80 µM but fell to below detectable levels by day 10 of growth arrest and cells started to lose viability at this time (Fig. 5B). Remaining viable cells accumulated high levels of GTP (1 mM), likely reflecting that enzyme activities for GTP synthesis and salvage were elevated because little to no ppGpp was present to inhibit them (20–22). The drop in ppGpp in aerobic *R. palustris* is like the dynamics of (p)ppGpp decline that is seen in *E. coli* relatively early in stationary phase (23). In *E. coli*, as in *R. palustris*, this decline in ppGpp is correlated with cell death. In *R. palustris* the loss in cell viability was paralleled by a drop in intracellular ATP. This is expected because cells lack a carbon and energy source required for ATP generation.

### Influence of ppGpp on gene expression in growth arrested cells

**Fig 6:**
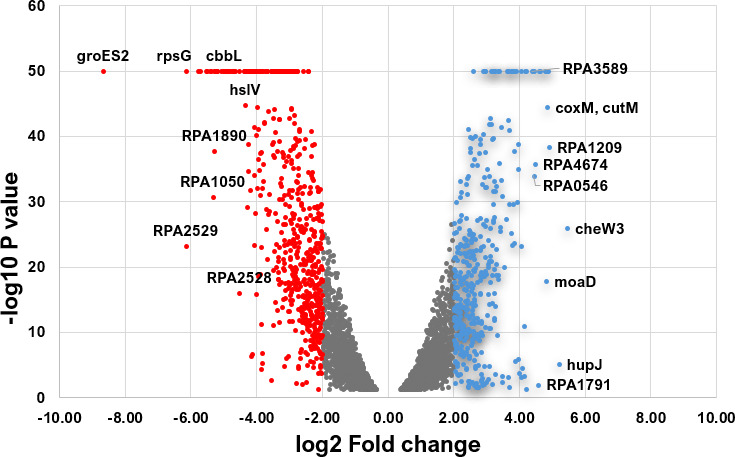
Volcano plot of RNA-seq comparison of WT and the ppGpp° strain at D1 postgrowth arrest. Each point represents a gene that is differentially expressed in an RNA-seq analysis that compared WT and the ppGpp° strain of *R. palustris* at D1 growth arrest with an adjusted *P*-value equal to or lower than ≤0.05 by deseq (26). Genes that are significantly upregulated (adjusted *P*-value   ≤ 0.05, log2 fold change  >  2) are represented with blue dots in the right side of the volcano plot. Genes that are significantly downregulated (*P* adjusted value ≤0.05, log2 fold change < −2) are represented as red dots in the left side of the volcano plot. In our DESeq2 analysis a collection of genes had a calculated *P*-value of 0, meaning the value was extremely small due to large consistent fold changes. For visualization, we have replaced these zeros with the small −log 10 *P*-value of 50.

Transcription is a major target of ppGpp in *E. coli* where it binds to two specific sites that are conserved in proteobacterial RNA polymerases, including that of *R. palustris* (24, 25). To evaluate the effect of ppGpp on gene expression in *R. palustris* growth-arrested cells, we compared the transcriptomes of WT and ppGpp° strains at D1 post-growth arrest. Cells are transcriptionally active at D1 and past work has shown that most of the major changes in WT strain gene expression that occur in growth arrest have happened by D1 (10). The effects of ppGpp on transcription at D1 were extensive, with the expression levels of over 65% of the approximately 4,836 protein-encoding genes in the *R. palustris* genome influenced by this nucleotide (*P*-value ≤ 0.05). The expression of 570 genes was ≤−4-fold lower in WT, ppGpp-replete cells compared to ppGpp° cells, suggesting that ppGpp played a direct or indirect role in repressing expression of this gene set. Another 394 genes were expressed at ≥4-fold higher levels in WT compared to ppGpp cells and thus activated by ppGpp at D1 growth arrest (Fig. 6).

We know that *R. palustris* continues to synthesize proteins in growth arrest but with reduced numbers of ribosomes (11). Consistent with this, and in keeping with observations with other bacteria, genes required for protein synthesis were a prominent target of transcriptional repression by ppGpp. About 40 ribosome protein genes were downregulated 4-fold or more by ppGpp and *fusA*, encoding elongation factor G required for translation, which was repressed almost 8-fold (Table S1).

Genes for protein degradation and folding were downregulated in ppGpp-replete cells at D1. These include *hslVU* and *clpXP* genes encoding ATP-dependent proteases and molecular chaperone genes *groES1*, *groES2*, *htpG*, and *dnaK*, which have various functions in folding or refolding of proteins and in preventing their aggregation (Table S1).

A plot of the *R. palustris* genes that changed in expression in response to ppGpp at D1 is shown in Fig. 6. In addition to *groES2*, *rpsG* (encoding 30S ribosomal protein S7), and *hslV*; the *cbbL* gene encoding the large subunit of ribulose bisphosphate carboxylase, the key enzyme of carbon dioxide fixation was downregulated in ppGpp-replete cells. Not labeled in Fig. 6, is the gene for the small subunit of this enzyme *cbbS*, which was repressed by ppGpp to a similar degree as *cbbL*. Repression of carbon dioxide fixation in growth arrest makes sense because cells are relatively quiescent and likely not generating much CO_2_ as a by-product of their metabolism. We labeled with their gene names four additional genes that we chose at random that were downregulated by ppGpp. All these genes are annotated as encoding hypothetical proteins (RPA1890, RPA2528, and RPA2529) and in the case of RPA1050, a hypothetical protein with a domain of unknown function (DUF3096). Genes RPA2528 and RPA2529 appear to be in an operon that may encode a bacteriophage. Of the 100 genes that were most highly downregulated by ppGpp at D1, 44 are genes of unknown function.

When we look at nine genes picked at random and labeled in Fig. 6 that were upregulated by ppGpp, there are four that have annotated functions. The *coxM*, *cutM* gene (RPA4668) is annotated as xanthine dehydrogenase family protein subunit M. Genes encoding the other two subunits (RPA4666 and RPA4667) of this predicted three-subunit enzyme were also upregulated by ppGpp (Table S1). Xanthine dehydrogenase family members are molybdopterin-dependent oxidoreductases. *R. palustris* encodes several of these enzymes and the functions of most of them are unknown. We were interested to see that *moaD* (RPA1169), encoding subunit 1 of the enzyme that catalyzes the last step in molybdopterin synthesis, was also upregulated by ppGpp in our experiments as was subunit 2 (*moaE* and RPA1168) of this two-subunit enzyme. Known genes that were upregulated by ppGpp at D1 are genes for uptake hydrogenase (one of the subunit genes, *hupJ* is indicated in Fig. 6). This enzyme functions to convert hydrogen gas to electrons for carbon dioxide fixation. Although the genes for carbon dioxide fixation are downregulated by ppGpp, *cbbR*, ending the transcriptional regulator that induces the carbon dioxide fixation operon in response to CO_2_ was upregulated over eightfold by ppGpp. Thus, ppGpp may prime cells in growth arrest to be ready for photoautotrophic growth. Two major categories of genes that were upregulated by ppGpp are chemotaxis genes, represented by CheW3, and transport genes, represented by RPA1791, which encodes a subunit of an ABC transport system (Fig. 6). *R. palustris* has three complete chemotaxis gene operons, all of which are expressed at higher levels in ppGpp-replete, growth-arrested cells. Genes annotated as encoding transport proteins comprise about 11% of the genes upregulated by fourfold or more by ppGpp at D1 compared to 2% of transport genes downregulated fourfold or more. An additional gene of interest is RPA0376. Upregulated over eightfold by ppGpp, RPA0376 encodes L-isoaspartate-O-methyltransferase (PIMT), an enzyme found universally in living organisms that repairs isoaspartates that accumulate spontaneously from L-asparagine and L-aspartate in aged proteins (27). The *R. palustris* enzyme has PIMT activity, but the relationship of this enzyme to cellular longevity has not been established (28). There were fewer genes, only 14, annotated as hypothetical or with a DUF domain among the top 100 genes most highly upregulated by ppGpp compared to the corresponding downregulated gene set (Table S1).

## DISCUSSION

This study suggests that sustained ppGpp production is important for *R. palustris* longevity over long periods of growth arrest in ATP-replete conditions. In our experiments, ppGpp, GTP, and ATP were produced by *R. palustris* at relatively constant levels over a prolonged period of 60 days of growth arrest. Our transcriptome results and data on posttranslational effects of ppGpp in other bacteria suggest that ppGpp enables essential processes like protein synthesis and protein turnover to proceed in nongrowing cells at reduced levels that allow for the turnover of cellular components at rates appropriate to the needs of *R. palustris* to sustain cellular integrity for long-term viability and to put it in a position to continue to forage for nutrients (Fig. 6; Table S1). An additional effect of ppGpp that we have not so far mentioned is to bind to GTPases associated with ribosome biogenesis and translation, including the GTPase Era, to modulate their activities (13, 29, 30). In past work, we have characterized Era (RPA2698) and YbeY (RPA0446), an rRNA maturation RNase that Era interacts with, as longevity proteins that are each required for 16S RNA processing and normal ribosome assembly in *R. palustris* growth-arrested cells, but that can be dispensed with in growing cells. It is likely that Era activity, though essential for longevity, needs to be partially inhibited by ppGpp in growth-arrested *R. palustris* to help ensure reduced rates of translation.

Our observation that the ppGpp° strain had highly elevated levels of GTP in growth arrest is consistent with studies in other bacteria showing that ppGpp inhibits key enzymes of purine synthesis and salvage that are also found in *R. palustris* (19, 20, 22). Dysregulation of GTP synthesis has been linked to the death of a ppGpp° strain of *Bacillus subtilis* (21), and this may be the proximal cause of death of the *R. palustris* ppGpp° strain in growth arrest.

ppGpp production in stationary phase has been characterized as a stress response in *E. coli*, but we did not see much evidence of this in *R. palustris*. For example, heat shock proteins were downregulated by ppGpp in *R. palustris*, not upregulated as in *E. coli. R. palustris* does not have a homolog of the stress sigma factor RpoS that is induced by ppGpp in *E. coli* and other gamma-proteobacteria (31), but it does have the alternative RNA polymerase sigma factor EcfG, considered to be the general stress response sigma factor of alphaproteobacteria (32, 33). The *ecfG* gene (RPA4225) was upregulated about sevenfold between log phase and D1 growth-arrested *R. palustris*, but its expression levels were influenced only about twofold by ppGpp in growth arrest. It will be interesting to examine the effects of EcfG on gene expression in non-growing cells and to see if the expression of any of the genes that it controls is also controlled by ppGpp.

In addition to N-terminal hydrolase (HD) and synthase (SYN) domains that direct the degradation and synthesis of ppGpp, RSH proteins have a C-terminal regulatory domain that has been less well characterized (18). We were surprised to find that a strain expressing a C-terminal truncated version of *rsh_rp_* behaved similarly to the WT in growth arrest (Fig. 3). This strain did not lose viability, and it had similar intracellular levels of ATP and GTP, and only slightly lower levels of ppGpp compared to the WT. This is at odds with a study in which *E. coli* expressing a *Streptococcus equisimilis* C-terminal truncated *rsh* gene had elevated ppGpp synthase activity (34). The conditions of our growth arrest experiments are relatively benign and involve the gradually imposed stress of carbon depletion. It may be that the C-terminal *rsh_rp_* deletion mutant would not survive an alternative type of starvation such as nitrogen or phosphate starvation or a nutrient depletion coupled with another stress, as effectively as the WT.

## MATERIALS AND METHODS

### Bacterial strains, growth, and incubation conditions

*Rhodopseudomonas palustris* CGA009 was used as the wild-type strain for this study. In-frame deletions of portions of the *R. palustris* RelA/SpoT gene *rsh_rp_* (RPA2693) were created using the suicide vector pJQ200SK, as described previously (11, 35). A Δ*rsh_rp_*64–192 (ppGpp^0^) mutant was constructed previously (11) by deleting the sequence encoding amino acids 64–192 of Rsh*_rp_* (Fig. 2). An additional mutant with the C-terminal regulatory domain deleted from *rsh_rp_* (Δ*rsh_rp_*407–760) was constructed for this study by deleting amino acids 407–760 of Rsh*_rp_* (Fig. 2).

Anaerobic cultures were grown at 30°C under constant illumination in rubber-stoppered Balch-type glass tubes in defined photosynthetic medium (PM) (36) amended with 20 mM sodium acetate as carbon source. Once cells entered growth arrest due to carbon depletion, cells were maintained in a 30° incubator with constant light. Aerobic cultures were grown in Erlenmeyer flasks in PM amended with 20 mM sodium acetate as the carbon source at 30°C in the dark. The cultures were stirred at 200 RPM. Cell viabilities were determined by plating serial dilutions on CA agar plates amended with 20 mM sodium acetate (16).

### Quantification of nucleotides

Bacterial nucleotides (ATP, GTP, and ppGpp) were quantified using an ultra-high performance liquid chromatography–tandem mass spectrometry (UHPLC-MS/MS) approach modified from Varik et al. (37). At each timepoint, liquid cultures were passed through a 0.2 μm (*d* = 0.47 mm) cellulose acetate membrane filter (Sartorius Biotech) using vacuum filtration. Cells were rinsed with 10 mL HPLC-grade water and immediately transferred to 50 mL conical tubes. Cells were washed off filters using 1 mL ice-cold 1 M acetic acid. The acetic acid-cell mixture was transferred to cryogenic vials, snap-frozen using liquid nitrogen, and stored at −80°C. Cold acid nucleotide extractions were performed by thawing samples on ice for 60 min with occasional vortexing. Samples were then re-frozen using liquid nitrogen and lyophilized for 6 hours using a VirTis Benchtop Freeze Dryer. Lyophilized samples were dissolved in 200 µL ice-cold HPLC-grade water, centrifuged at maximum speed for 30 min, and clear supernatant was collected for quantification via UHPLC-MS/MS. UHPLC-MS/MS was performed using an ACQUITY Premier UPLC System coupled with a Waters XEVO TQ-S triple quadrupole mass spectrometer as described (23). Intracellular concentrations of nucleotides were determined by normalizing measured concentrations to cell numbers (CFU determined by plating) and the estimated average size of cells in growth arrest obtained as described previously (11).

### RNA-seq analysis

*R. palustris* CGA009 wild type and Δ*rsh_rp_*64–192 (ppGpp^0^) cells were grown in light under anaerobic conditions in PM medium with acetate as the carbon source, as described above. Samples from the mid-logarithmic phase of growth were collected at OD_660_ = ~0.6. After cultures stopped growing due to carbon depletion, samples were taken at D1 and D6 of growth arrest. Biological duplicates were treated as described previously (10). RNA was extracted from 5 mL samples using the miRNAeasy minikit (Qiagen), treated with TURBO DNase (Ambion), and purified with RNeasy MinElute Cleanup kit (Qiagen). The samples were then sent to Genewiz, Inc., for library preparation and HiSeq RNA-seq sequencing. Raw RNA-seq reads were quality filtered and trimmed of adapters with Trimmomatic v0.39 (38) and the following parameter settings: HEADCROP:15 LEADING:3 TRAILING:3 SLIDINGWINDOW:4:15 MINLEN:35. Surviving read quality was assessed with FastQC (Babraham Bioinformatics). Reads were aligned to the *R. palustris* CGA009 reference genome and residual rRNA/tRNA reads were removed using Strand NGS v4.0, build 242089 (Strand Life Sciences) following default parameters. Differential gene expression analysis was performed using DESeq2 (fold change ≥ 2, *P*  ≤ 0.05) (26). Sequencing results were processed and analyzed in-house with StrandNGS (strand-ngs.com). Wild-type RNA-seq data were previously reported in (10).

### Genome sequence

The version of the *R. palustris* CGA009 genome used for our RNA-seq analysis and referred to in Table S1 is the National Center for Biotechnology Information (NCBI) Reference Sequence NC_005296. This has recently been replaced by a resequenced and reannotated *R. palustris* CGA009 genome with new locus tags (39). The newer version of the genome is now available as NCBI Reference Sequence: NZ_CP116810.1. Table S2 cross-references the two sets of locus tags and provides the updated gene annotations from NZ_CP116810.1. We manually replaced the original gene annotations for the 100 genes most up-regulated by ppGpp and for the 100 genes most downregulated by ppGpp in Table S1 with the updated annotations from Table S2.

## Data Availability

All processed data are supplied as Table S1. Raw data are available on request from the corresponding author.

## SUPPLEMENTAL MATERIAL

Captions mbio.01079-26-s0001.pdf Supplemental table captions.

Table S1 mbio.01079-26-s0002.xlsx RNA-seq data comparing *R. palustris* WT and ppGpp° strains at D1 growth arrest.

Table S2 mbio.01079-26-s0003.xlsx Comparisons of locus tags for two different annotated versions of *R. palustris* CGA009.

ASM does not own the copyrights to Supplemental Material that may be linked to, or accessed through, an article. The authors have granted ASM a non-exclusive, world-wide license to publish the Supplemental Material files. Please contact the corresponding author directly for reuse.
