# Supplementary material for: Sustained ppGpp production underpins months-long survival of a bacterium in growth arrest
Source: mBio. 2026 Jun 18;17(7):e01079-26. doi: 10.1128/mbio.01079-26 (PMC13343836; doi:10.1128/mbio.01079-26)
Supplement: Captions — Supplemental table captions. [file mbio.01079-26-s0001.pdf]

Supplemental Table Legends.

Table S1. Analysis of RNA-seq data of *R. palustris* WT D1 vs. ppGpp° D1 cells Log2 Fold change.

Table S2. Locus tags of *R. palustris* CGA009 NCBI Reference Sequence NC_005296 compared to the renamed locus tags of the reannotated *R. palustris* CGA009 NCBI Reference Sequence: NZ_CP116810.1.
